# Systemic signalling and local effectors in developmental stability, body symmetry, and size

**DOI:** 10.15698/cst2018.12.167

**Published:** 2018-11-13

**Authors:** Sergio Juarez-Carreño, Javier Morante, Maria Dominguez

**Affiliations:** 1 Instituto de Neurociencias, Consejo Superior de Investigaciones Científicas-Universidad Miguel Hernández (CSIC-UMH), Avda Santiago Ramón y Cajal s/n, Campus de Sant Joan, Alicante, Spain.

**Keywords:** robustness, fluctuating asymmetry, environmental stress, somatic mutations, developmental stability, buffering, neuroendocrine signaling, Dilp8, Lgr3, relaxin, IGF

## Abstract

Symmetric growth and the origins of fluctuating asymmetry are unresolved phenomena of biology. Small, and sometimes noticeable, deviations from perfect bilateral symmetry reflect the vulnerability of development to perturbations. The degree of asymmetry is related to the magnitude of the perturbations and the ability of an individual to cope with them. As the left and right sides of an individual were presumed to be genetically identical, deviations of symmetry were traditionally attributed to non-genetic effects such as environmental and developmental noise. In this review, we draw attention to other possible sources of variability, especially to somatic mutations and transposons. Mutations are a major source of phenotypic variability and recent genomic data have highlighted somatic mutations as ubiquitous, even in phenotypically normal individuals. We discuss the importance of factors that are responsible for buffering and stabilizing the genome and for maintaining size robustness and quality through elimination of less-fit or damaged cells. However, the important question that arises from these studies is whether this self-correcting capacity and intrinsic organ size controls are sufficient to explain how symmetric structures can reach an identical size and shape. Indeed, recent discoveries in the fruit fly have uncovered a conserved hormone of the insulin/IGF/relaxin family, Dilp8, that is responsible for stabilizing body size and symmetry in the face of growth perturbations. Dilp8 alarm signals periphery growth status to the brain, where it acts on its receptor Lgr3. Loss of Dilp8-Lgr3 signaling renders flies incapable of detecting growth perturbations and thus maintaining a stable size and symmetry. These findings help to understand how size and symmetry of somatic tissues remain undeterred in noisy environments, after injury or illnesses, and in the presence of accumulated somatic mutations.

## INTRODUCTION

Most animals, including humans, exhibit bilateral symmetry. This symmetry refers to the external body plan, as internal visceral organs are often positioned asymmetrically with the left and right sides falling under a different genetic control. Bilateral symmetry means that although each side of a body grows separately they manage to produce identical halves. Attaining such symmetry is extremely important not only for the overall balance and coordination of the body, but also for the high performance of specific body parts such as hips and legs, the jaw, or the wings of an insect. At first glance, symmetry is deceptively simple. In fact, the perception outside this field is that bilateral symmetry arises naturally because the two sides are genetically identical. However, the rules and genetic processes underlying such high-order control of growth appear to be genetically complex [1–3] and remain elusive until recently.

**Figure 1:**
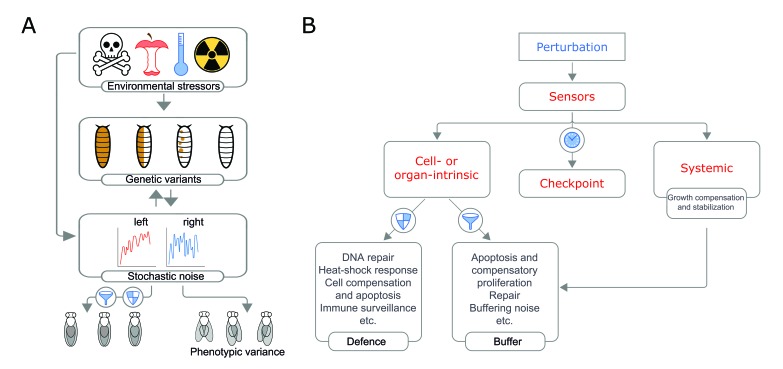
FIGURE 1: A speculative model of the three sources of perturbation and their interactions in generating intra- and interindividual phenotypic variability, illustrated in flies. **(A)** *Drosophila* larvae may acquire mutations that can be expressed in a mosaic state (brown dots) or inherited from one of the parents (all brown). Stochastic noise may cause gene expression fluctuations and variability in growth between the left and right sides. Environmental factors may cause changes in gene expression by genetic and non-genetic effects. Biological noise can cause replication errors that result in mutations e.g. [61] and thus it may also contribute to somatic mutations. **(B)** Developmental robustness requires mechanisms that sense damage and growth perturbations inflicted at the cell, organ, and systemic level. Specific damage sensors activate coordinated responses that trigger checkpoints such as transcriptional or cell cycle arrest to provide time for repair or to counterbalance perturbations (e.g., during a thermal stress). When damage is unrepairable, cellular stress sensors also initiates the apoptosis of the damaged cells. To maintain tissue growth and homeostasis, stress sensors such as p53 trigger compensatory proliferation. Massive tissue damage activates local regenerative responses and trigger a checkpoint that delays developmental timing (maturation) and act systemically. In addition, a variety of surveillance mechanisms maintain tissue quality control by detecting and eliminating both less fit and potentially harmful cells.

Symmetry is never perfect; we all have some subtle differences between the left and right sides of our body. For instance, our two feet can be slightly different sizes and most of us perceive one side of our face/body as being more beautiful than the other. These mismatches are a consequence of environmental factors, errors in development (noise) and mutations (illustrated in **Figure 1**). The degree of deviation from perfect bilateral symmetry reflects both the magnitude of these disturbances [4] and an individual's ability to cope with (suppress) the effects during growth [5, 6]. Bilateral symmetry is not, or not only, an aesthetic problem. In some children, facial and body asymmetries are more evident, which, along with a delayed or irregular growth rate, are indicative of a possible underlying genetic condition. Regular growth is one of the best indicators of a child's general health and also acts as a predictor for health in adult life [7]. Importantly, children, like juveniles of other animals, have a remarkable plasticity that enables them to recover from illnesses, starvation, and injuries that delay or deviate normal growth trajectory, and attain the correct target size and perfect bilateral symmetry [8, 9]. This supports that body size is 'canalized' [10] i.e. meaning highly robust and buffered against variability in the environment or genetics (Waddington's canalization, [11]).

The development of a human body involves trillions of cell divisions and years of growth in naturally fluctuating environments, with recent genomic data highlighting that organisms accumulate a substantial number of mutations in their somatic cells during replication [12]. It is astonishing that most individuals have a nearly perfect symmetry. These observations suggest that somatic cells are able to endure stochastic perturbations and mutations that may result from environmental factor-induced DNA damage, transposon mobilization and inaccurate DNA replication or repair [13] and still maintain size stability and symmetry.

**Figure 2:**
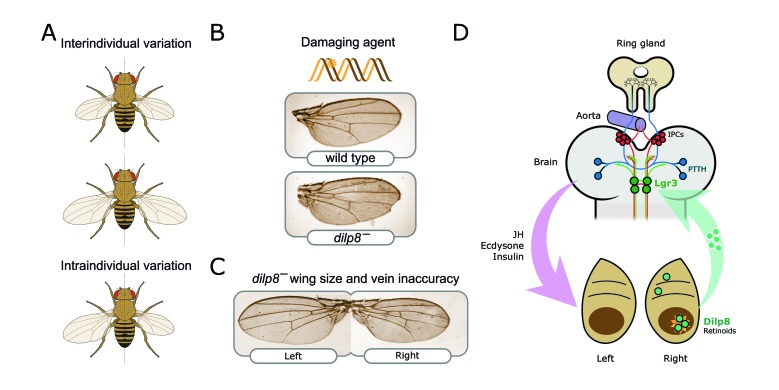
FIGURE 2: Neuroendocrine control of symmetry and body size. **(A)** Loss of the Dilp8 hormone yields flies with variable body sizes (inter-individual) and increased bilateral asymmetry (intraindividual variability). **(B)** Feeding *Drosophila* juveniles with the DNA-damaging agent ethyl methanosulfonate (EMS) induces massive cell death in the imaginal discs, cell cycle arrest, and strong developmental delay. Without *dilp8* (bottom wing), EMS-fed animals cannot recover from this damage and exhibit a 6-fold increase in pattern and growth inaccuracies [14]. **(C)** *dilp8* mutants exhibit left-right wing asymmetry and also pattern inaccuracies, which may reflect the unmasking of pre-existing or acquired mutations, stochastic noise, and/or the negative effect of the environment (e.g., temperature stress). **(D)** Dilp8 produced by damaged or growth-perturbed cells, which also activates the production of other ‘alarm' signals such as retinoid signals [145] is released to circulation and acts in the brain through the relaxin receptor Lgr3 (green neurons). Lgr3 co-regulates two neuronal populations which control growth and maturation rate by acting on the ring gland. The ring gland is a central neuroendocrine organ regulating organismal growth rate and timing of maturation [45]. Distinct groups of cells within the ring gland — the corpus allatum — produce the juvenile hormone (JH), and cells of the prothoracic gland synthesise and release the steroid hormone ecdysone. Its complex functions are centrally controlled by neurons that produce the prothoracicotropic hormone (PTTH; represented as blue circles) [139,146] and the insulin-producing cells (IPCs; red circles). IPCs produce insulin-like peptides, primarily Dilp2, Dilp3, and Dilp5, and regulate systemic growth and ecdysone biosynthesis (reviewed in [47].

But how exactly do both halves of a human face and/or body end up being equal? Recent work has shown that the robustness of body size and symmetry in flies involves extensive communication under the control of the central nervous system via the hormone Dilp8 (Drosophila insulin/IGF/relaxin-like peptide 8) [14]. While Dilp8 is produced by peripheral tissues in response to growth perturbations [14, 15], this 'alarm' signal acts on a receptor (relaxin Lgr3) in the brain [16–18]. Loss of *dilp8* [14,19] or of its receptor *lgr3* in neurons [16–18] renders flies incapable of maintaining a strict control over their size, resulting in flies with highly variable body size and, intra-individually, with disproportionate growth and left-right asymmetry (illustrated in **Figure 2**). These discoveries may provide support for the neuroendocrine or '*sizostat*' hypothesis of Tanner (1963), who suggested that feedback endocrine signalling enables the brain to recognize a mismatch in growth, and thus adjusts the growth rate accordingly to the degree of mismatch [20]. Recent experimental evidence in mice has further provided evidence for communication and systemic signalling in the stabilization of growth and maintenance of symmetry in the face of perturbations [21, 22].

In this review, we attempt to provide an alternative account of developmental stability and precision in size regulation by considering local and systemic mechanisms and possible genetic origin (i.e. somatic mutations) of asymmetry. Although cells have surveillance and editing systems that ensure DNA fidelity is maintained during replication, stress or damage can overwhelm these systems and ultimately can result in mutations and disease states like cancer [23, 24]. How cells and multicellular organisms buffer genomic variation to ensure uniform phenotypic outcomes has been extensively investigated by population geneticists, evolutionary geneticists and systems biologists, often in relation to the concept of evolvability [25, 26]. However, developmental buffering masks the fact that organisms continuously accumulate genetic mutations, not only in small clones of germline cells but also in somatic tissues. Loss of buffering unmasks these hidden genetic variants and may result in the manifestation of non-constant phenotypes or disease processes such as sporadic cancer.

Somatic mutations can result from radiation, chemicals, viruses, and other environmental factors that damage the DNA, transposon mobilization, and inaccurate DNA replication [27, 28]. Furthermore, it is known that individuals are more prone to such errors and transposon-induced mutations during early development [23, 29] than at any other stage of life. This is highly relevant because mutations that arise during early development will be present in a larger fraction of cells within the organism and may even occupy a whole organ or half of the body. As such, we attempt to convey a more comprehensive view of developmental precision, one that takes into account the tactics used to scape or avoid perturbations both at the cellular and organism-wide level and that are responsible for stabilizing genome and for maintaining correct organ size and quality through eliminating less-fit or damaged cells. We also review our current view of strategies and factors for buffering the effects of unavoidable variations. Although we concentrate on studies examining the wing imaginal discs of *Drosophila*, many of the principles behind body symmetry and size regulation are likely conserved. For further discussions of developmental buffering, canalization and other paradigms see also [9, 30–32].

## TWO MATCHING SIDES: THE PROBLEM OF SYMMETRIC GROWTH

The ability to produce a constant and invariant phenotype despite perturbations is a widespread but poorly understood property of living organisms. Clinical and animal studies have demonstrated that children and juveniles of most animal species have a remarkable capacity to recover and regain their normal size after illnesses, temporary malnutrition, infectious diseases and other adverse conditions. Although this demonstrates that body size is ‘canalized' [10] —the concept that refers to the property of an individual or an individual trait to produce a constant phenotype despite genetic and environmental differences [11], the underlying genetic mechanisms have remained a mystery, or at least until recently.

Robust body size and symmetry entail high-order control of growth and precision during development. However, to date, studies of growth regulation have barely addressed the mechanisms behind this phenomenon. As we discuss below, consistency in organ size and symmetry (both inter and intra-individually) requires not only robust developmental programmes but also requires extensive communication between growing organs and the brain and strategies and pathways that detect mismatches and correct, repair and stabilize size against perturbations.

Given that recent data have highlighted the extensive existence of hidden genetic variations (often in the mosaic state) in phenotypically normal individuals [12], here we consider that bilateral asymmetry can have a genetic origin [13]. Moreover, we argue that different types of growth perturbations (e.g., mutational, environmental, noise, or accidents) may be stabilized by similar and overlapping mechanisms, and that both fluctuating asymmetry and more conspicuous differences — including those seen in several human growth disorders [33] — may represent a continuum of the same self-stabilization process, reflecting different sensitivities to and/or magnitudes of the perturbations.

The size of the body and its parts (e.g., limbs) are also strongly responsive to certain environmental factors, particularly nutrition and changes in temperature [34, 36], resulting in animal with smaller size than well-fed or bread sibling under unstressed conditions. This level of size plasticity enables developing organisms to cope with certain environmental variability (temporal and/or spatial), maintaining fitness under new conditions [37, 38]. However, such plasticity in size and shape (e.g. producing smaller cells and animals in starvation conditions) can sometimes be maladaptive and negatively impact performance and fitness [39]. As such, apart from the clear advantage of maintaining symmetry [5], children and juveniles of other animals tend to defend their target body size when variations can be harmful [8, 10]. Thus, understanding how a robust body size and symmetry is achieved and how cells and organisms remain undeterred despite significant biological noise, environmental and genomic variation could have broad implications for fitness and health.

## THE SYMMETRY OF INSECT WINGS: A MODEL FOR DEVELOPMENTAL PRECISION AND STABILITY

The symmetry of insect wings is a convenient paradigm for investigating the resilience of organisms to mutational and environmental influences. The symmetry of insect wings is highly robust e.g. [40], yet at the same time, sensitive to perturbations e.g. [14, 41]. As fly wings are flat structures, they can easily be measured in an accurate manner [42, 43]. This is important because bilateral asymmetry, although significant, can be subtle and difficult to assess without accurate measurement methods [44]. Moreover, final wing size is not altered by mechanical use, and thus, asymmetry can be unequivocally attributed to perturbations during growth.

Just like most external parts of the adult *Drosophila melanogaster*, the wings develop from imaginal discs that grow inside the larva. Each larva has nine bilateral pairs of imaginal discs plus a single genital disc. Several recent reviews discuss extensively the mechanisms and genes that regulate regulate growth and recovery in wing imaginal disc [45–47] and in mammals [9, 48]. We thus focus on the mechanisms that may ensure robustness in size control and symmetry.

Symmetry between insect wings, just like symmetry between vertebrate limbs (another useful system for studying symmetry) [9, 22, 49, 50] emerges even despite vast differences in cell proliferation and growth rates between the two sides [4, 50, 51]. Imaginal discs proliferate exponentially over the course of four days and increase their mass by approximately 1,000-fold before differentiating into adult wings upon metamorphosis. Achieving a proper final size and shape depends on genes involved in patterning, specification of organ and segment identity, cell polarity and control of cell proliferation, and cell growth [52], hormones and nutrient-sensing pathways [45, 47], and mechanical cues and/or tension as organ grow [53], as reviewed in [45, 46]. Programmed cell death is also important as it helps fine-tune final size and shape, both during and after cell differentiation during metamorphosis [54].

Due to a high level of regulation, the growth of imaginal discs is robust against perturbations. For example, although radiation and DNA-damaging agents can kill up to 75% of the cells in the imaginal discs, growth becomes reactivated so that nearly normal adult flies are formed e.g. [55]. Furthermore, inhibition of cell division in one part of an imaginal disc is readily compensated by changes in cell size. Such compensation results in overall normally sized parts with fewer but larger cells (reviewed in [56]). Broadly, compensation mechanism, regeneration, or the catch up growth after illnesses or starvation, entail mechanisms that detect growth mismatches, the magnitude of mismatch, and the source of perturbation. Three types of growth perturbations are discussed below, along with the ‘sensors' and the local and systemic factors that may enable organisms to remain undeterred against perturbations and developmental errors.

## SOURCES OF PERTURBATIONS AND PHENOTYPIC VARIABILITY

Variability in body size and asymmetry can be the result of many confluent factors and cumulative effects of successive perturbations (**Figure 1A**). The three primary sources of variability are inherent stochastic noise, the environment and genetic variation, which may arise from the unmasking of mutations acquired in somatic cells [13].

### Stochastic noise

Extensive work in yeast, mammalian cells, and *in vivo* in various model multicellular organisms has revealed that gene expression is exceptionally noisy [57–60]. This can pose a major challenge for generating consistent and reproducible phenotypes. Noise is defined as the observed stochastic variation in gene expression among isogenic cells under the same conditions. This explicitly relies on the assumption that isogenic cells, such as cells in cloned animals, monozygotic twins, and cells of symmetric traits within an individual, remain genetically identical through successive cell divisions over time. As Uphoff and colleagues (2016) point out, noise can reduce the capacity of cells to repair DNA and thereby cause cell-to-cell variation through mutations [61]. These observations broaden the potential causes of phenotypic variation and (potentially) asymmetry and highlight the importance of comprehensive genomic analysis of asymmetric traits. Noise however, is not always harmful, and natural stochastic fluctuations play an important role in various cell fate decisions within multicellular organisms and the phenotype of single cells. In this review, we focus on the negative effects that stochastic variation has on the execution of constant growth patterns in development.

Even though biological noise occurs at all levels — during transcription, translation, chromatin remodelling, and biochemical cascades — final phenotypes exhibit exceptional endurance to noise-driven variability [59]. Such robustness implies that noise is largely filtered out by the genetic network [24, 32]. Feedback [24, 51], autoregulatory loops [62], genomic redundancy (gene and pathway duplication, cis-regulatory and promoter redundancy) [64–67], redundancy in cell numbers [54], alternative or compensatory pathways [66], and distributed robustness [67] are systems strategies for filtering fluctuations in gene expression and protein concentration levels caused by environmental and genetic variation. In addition, several researchers have suggested that hubs (i.e., the most connected genes in gene networks) might be particularly important for the stabilization of development against perturbations e.g. [68]. Chromatin remodelling factors, chaperones, and microRNAs are examples of factors that contribute to buffering this source of variation (see section on “Buffering”).

### Environmental stressors

The environment can significantly impact development, particularly early in life. For example, a higher degree of asymmetric individuals is consistently associated with stressed marginal habitats [4, 5] and areas prone to industrial pollution [7]. Laboratory studies have also shown that compared to the progeny of unstressed control rats, the progeny of rats inflicted with stress tend to be more asymmetric [69].

Temperature variation challenges the ability of an organism to cope with perturbations and while temperature increases typically result in a higher degree of fluctuating asymmetry [4], the underlying molecular mechanisms are rarely discussed. Chen and Schloetterer (2015) used genetically different strains of *D. melanogaster* to analyse gene expression dynamics at four different temperatures [70]. At 18°C, the different *D. melanogaster* strains exhibited very similar gene expression levels and a constant phenotype despite their extensive genetic variability. At higher temperatures (27-29°C) however, high levels of phenotypic variance and large variations in gene expression were detected between the different genetic strains. This indicates that the strong buffering of gene expression breaks down as temperatures increase [70]. This, together with other evidence, supported the idea that environmental stress can cause phenotypic variation by unmasking hidden (meaning pre-existing and silent) genetic variants and enhancing stochastic gene expression variation.

Physical accidents, infectious diseases, and malnutrition can also disturb growth, both locally and globally [71], and evidence in flies suggests that these sources of variability are suppressed by a common mechanism that also buffers small, random noise [14]. It is also known that numerous environmental substances and chemicals can disrupt endocrine function and cause stunted growth in children [72]. Furthermore, environmental factors can also cause changes directly to the DNA (see below).

### Somatic mutations and transposable elements

Mutations are the main source of phenotypic variability. Somatic mutations can result from environmental factor-induced (e.g., radiation, viruses, temperature stress, natural and man-made substances such as pollutants, pesticides) DNA damage and inaccurate DNA replication [27] that accumulate in germline and somatic cells throughout an organism's life [12].

DNA is also continuously challenged by endogenous by-products of cellular metabolism (e.g., reactive oxygen species; ROS) [73] and transposon activity [28]. As first described in the early 1950 by Margaret McClintock in *Zea mays*, transposons are now known to be a major source of somatic mosaicism and phenotypic diversity (revised in [74]. Furthermore, it is known that individuals are more prone to such errors and susceptible to stress-induced mutations during early development [29, 75].

Recent data from mice and humans highlight that the mutational rate in somatic cells is two to ten orders of magnitude higher than the rate in germline cells [76]. In flies, the rate of somatic mutations is even higher (3-fold higher than in mice) [77] and mainly result from genome rearrangements [77]. In addition, temperature significantly increases the rate of new somatic mutations in flies [65] and, during aging, the accumulation of frequent genomic alterations and loss of heterozygosity (LOH) as first described in the 1930's [78] causes intestinal neoplasia in adult flies as seen in humans ([79] and citations therein). Recent structural genetic analysis in flies, humans and other animal species underscores the presence of extensive somatic copy number variations even in apparently phenotypically normal individuals (e.g., [80–82]). Studies in flies suggest that compensatory mechanisms and genetic heterozygosity largely mask the effects of such copy number variations [82], which seems to clarify the link between inbreeding and increased fluctuating asymmetry described in earlier studies [83].

While somatic mutations are not inherited by offspring, they can contribute to intraindividual variability and disease processes [84]. That is, the cause of fluctuating asymmetry may be genetic in origin even though the variability is not heritable. The left and right sides of an individual, like monozygotic twins, cloned animals and monoclonal cell populations have typically been assumed to comprise identical genomes. Therefore, discordance in monozygotic twins and deviation from bilateral symmetry, for example, have been ascribed to non-genetic effects arising from environmental influences and/or the stochasticity of biological processes [59, 85, 86]. However, this view is challenged by genomic studies of concordant and discordant monozygotic twins underscore that even small variations in gene copy number may have large phenotypic consequences. While the role of *de novo* somatic genetic changes is well-characterized in cancer, less is known about their contribution to individual variability and predisposition to non-cancerous syndromes [84] and (**Figure 1A**).

Some overgrowth syndromes provide further suggestion that mosaicism for certain mutations may underlie left-right abnormalities. Facial, trunk and/or limb asymmetry are clinical manifestations of numerous overgrowth syndromes, including the Proteus, Pallister–Killian, and Beckwith-Wiedemann syndromes [33, 87–89]. Many cases are caused by *de novo* somatic activating mutations, or imprinted defects, affecting the same signalling pathway, the insulin-like growth factor-2 (IGF-2)/PI3K/AKT pathway [33,90]. This indicates that all these syndromes could be a spectrum of the same disease. Silver-Russell syndrome is another asymmetry syndrome but, in this case, it is associated with severe intrauterine and postnatal growth retardation, and consistently, is caused by imprinted defects resulting in loss of the IGF-2 pathway often manifested as somatic mutations [89]. Beckwith-Wiedemann and Silver-Russell syndromes are strikingly common syndromes in twins, but only the discordant twin develops the syndrome [91, 92], supporting that the genetic abnormality occurs post-zygotically. Altogether, these observations hint at a possible genetic basis for non-syndromic fluctuating asymmetry.

Importantly, these clinical data and recent empirical studies in flies and mice highlight the insulin/IGF/PI3K/Akt pathway as a common pathway in stabilization of growth e.g. [16, 22, 33]. IGF/PI3K/AKT pathway is a universal pathway that regulates cell and organismal growth in yeast, flies, worms, mice, and humans [9, 48, 93]. Future studies will determine the systemic and local actions of this pathway in developmental (in)stability and symmetric growth in humans and other animals.

## SENSING PERTURBATIONS, DAMAGE AND FITNESS IN GROWING ORGANS

Organisms have evolved multiple mechanisms to resist and withstand the harmful effects of perturbations during development and growth (**Figure 1B**). We highlight a few of these mechanisms to illustrate how continued proofreading and quality control maintain tissue growth and homeostasis to ensure correct organ size (see also for additional 'defence' mechanisms [23]. These defences act at the cell, organ and organism level.

### Cellular sensors and defences against perturbation

The genome of an organism is constantly damaged and changed. When this occurs within individual cells, cellular diversity is generated. The impact of such changes not only depends on the changes themselves but also on how they interact with environmental factors and intrinsic noise to produce phenotypic variability (illustrated in **Figure 1A**). Together, errors in replication and transposon mobilization induced damage appears to account for most of the mutations in our cells, including gross chromosomal rearrangements [27, 28]. Cells employ various conserved editing mechanisms to correct these mutational mistakes and maintain the fidelity of the DNA [27, 94]. DNA damage is sensed by specific sensors that activate repair pathways and trigger checkpoints to allow time for repairs. For example, a cell cycle arrest checkpoint can signal the presence of DNA damage and stall the replication fork until repair has been completed [95]. When damage is unrepairable, the stress sensor/transcription factor p53 coordinates the checkpoint with the initiation of apoptosis in severely damaged cells [96, 97] and restores tissue growth and homeostasis via a compensatory proliferation that replaces the lost cells [98].

Originally described in *Drosophila*, the heat-shock response is another fundamental protective mechanism because extreme temperature can cause severe damage to important cellular structures and DNA [99]. Even mild temperature increases have been found to result in morphological inaccuracies and fluctuating asymmetry in human and non-human vertebrates [5, 71]. Similarly, in flies, a mere increase of only two degrees Celsius significantly increases fluctuating asymmetry of wing size and shape [14, 16–18, 101], features which are normally highly robust [40]. Heat shock causes proteins to unfold and aggregate, and the unfolded proteins, not the temperature per se, activate the heat-shock response that protects the cell against the damaging effects of the abnormal proteins and aggregates. Errors in protein synthesis, which are five to six orders of magnitude more frequent than genetic mutations, can also lead to unfolded/misfolded proteins and phenotypic inaccuracy and also activate the conserved heat-shock response [102–104]. Molecular chaperones are thus essential components of quality control that aid in the folding and maintenance of newly translated proteins and thus, in the defence against heat stress. For example, Hsp90 is a key member of the heat-shock response and a conserved factor in buffering [105–107] (see discussion in ‘Buffering factors').

Misfolded/unfolded proteins accumulate in the endoplasmic reticulum (ER). This stresses the ER and can ultimately cause cell damage. In turn, cells activate a conserved unfolded protein response (UPR) that induces expression of UPR effectors to help cells cope [108]. The UPR primarily elicits its protective role through the activation of the Ire1/Xpb1, Atf6 and Perk pathways (reviewed in [108]). However, chronic ER stress can also trigger apoptosis via activation of the Jun-N-terminal kinase (JNK) pathway, and a developmental delay checkpoint via activation of the Dilp8 hormone [109]. Together, these pathways help maintain cell homeostasis and tissue integrity.

### Organ fitness, cell competition, growth compensation and symmetry

Multicellular organisms have a greater capacity to resist perturbations and survive compared to unicellular organisms partly because the injury or death of some cells does not affect the whole organism and the affected cells can be replaced. To maintain tissue health, cells in growing organs continuously monitor their growth, survival and fitness in relation to neighbouring cells [52, 110]. For instance, through cell competition, cells that are unfit or that have a growth disadvantage are actively eliminated by fitter neighbouring cells [111–113]. Before dying, less fit cells signal to neighbouring cells to trigger compensatory proliferation by activating the JNK pathway and producing mitogenic signals. This ultimately leads to a constant size [52]. Damaged and unfit cells are engulfed either by specific innate immune cells or by the fitter epidermal cells [114–116]. Cell competition is mediated by the Myc protooncogene [117, 118] and also involves activation of mitogens belonging to the Wingless/Wnt and BMP/Dpp families (for further discussions of factors, fitness signals and mechanisms of cell competition see [111, 119]). The innate immune system also play a role in maintaining the health of growing parts and tissues by eliminating apoptotic or sub-viable cells that are damaged. Failure to induce apoptosis causes erroneous morphogenesis [120]. This is illustrated with the death factor Hid, which upon deletion, results in an increased fluctuating asymmetry of wings and a high variability in wing size [52]. Although cell competition was first described in the *Drosophila* wing imaginal disc, several recent studies demonstrate that this quality control process also acts during early embryogenesis in mice and in adult stem cells (reviewed in [111]*.*

Compensatory growth and regeneration are used to replace damaged tissue during animal development and, when tissue damage is severe or extensive, the stress sensor p53 coordinates a cellular response that arrest the cell cycle, triggers apoptosis and a developmental delay checkpoint that postpones the larval–pupal transition (i.e., sexual maturation) until the tissue is recomposed by compensatory proliferation [98, 121, 122].

Tissue health against neoplastic insults is also controlled via activation of the stress response and pro-apoptotic JNK pathway by recognising and eliminating cells with ‘growth advantage' due to abnormal expression of mitogen and morphogenetic signals like Dpp [123]. This mechanism is complementary to cell competition and also involves specific innate immune defences [124–126] and involves different immune cells to fright off and reduce the potential risk of ‘preneoplastic' cells in organs and the organism as a whole. This defence mechanism is known as Epithelial Defence Against Cancer or EDAC.

Recent research has also shown that intra-organ needs to be coordinate to ensure proper organ shape via local and hormones signals. For example, growth perturbation in one part of an imaginal disc affects the growth of the unperturbed part. In such a scenario, a compensatory change in the wild type part is triggered so that it matches the growth of the perturbed compartment [127, 128]. This coordination is dependent on p53 in the perturbed part [127] and the activation of ecdysone and IGF signalling for cell-non-autonomous coordination [128]. This intra-organ adjustments and modulation of growth help to maintain organ proportion despite overall organ change.

The sensing and elimination of aberrant or damaged cells by neighbours and/or immune system cells relies on signals produced by the abnormal cells [119, 120, 129] via incompletely understood mechanisms. Mechanical stress is one such mechanism implicated in the regulation of organ size in insect imaginal discs, vertebrate limb bones, and also in plant organs [9, 46, 130]. In particular, mechanical stretching forces are thought to buffer variations in growth rate across organs and to help maintain organ size homeostasis [131, 132]. Mechanistically, there is also growing evidence that maintaining the balance of mechanical stress between neighbouring cells involves the activation of the Hippo pathway via the growth regulatory sensor Yorkie (Yki) and the mammalian YAP and TAZ growth effectors are also important [133].

These studies highlight the importance of sensors, surveillance, repair, and quality selection mechanisms during the development of body parts. The important question is whether this self-correcting capacity is sufficient to explain how symmetric structures like the limbs can reach an identical size and shape. Classical vertebrate limb and jaw transplantation experiments in hosts with different growth parameters showed that these structures tend to grow to approximate the size of the donor part [134, 135]. Is this approximation accurate enough to explain symmetry, or could extrinsic control also play an important role?

### Body and organ size-control: local or systemic?

The importance of systemic signals (hormones) for the canalization' of body size is demonstrated by stunted growth and gigantism — two conditions resulting from the abnormal production of brain-derived growth hormones. Thus, the brain plays a critical role in the maintenance of the correct regulation of body size by modulating hormones related to systemic growth control. Hormonal factors are also of paramount importance for catch-up growth after illnesses and injury in humans and other mammals [136].

Intuitively, the production of hormones that trigger maturation (i.e., end of linear growth) must be delayed until all organs have completed their growth. This developmental delay or checkpoint requires (1) specific sensors that recognize growth mismatches and (2) adjustments in the production of hormones regulating growth rate and developmental timing. In this way, an organism is able to recover from growth disturbances by delaying maturation in proportion to the amount of growth that still needs to be completed.

In the 1960s, Tanner [20] postulated that the ability of children to recover or catch up with their growth after illness, starvation, and other conditions that slow growth would involve a feedback inhibitory mechanism known as the neuroendocrine or *sizostat* hypothesis. The *sizostat* would consist of two elements: a factor produced in proportion to the mass of the organ, whose levels increase with time as the organ grows, and a receptor for that factor expressed in the cells of the *sizostat* in the nervous system, whose levels increase as the organism ages, though more slowly than the ligand [20] and reviewed in [9, 136]. In the model, the unbound receptors would serve as a trigger for the release of a growth-promoting hormone that regulates both normal growth and the catch-up growth phenomenon. After an injury or condition delaying growth, the concentration of the circulating signal produced by the slow growing, and thus smaller organs, would be lower than expected based on the organism's chronological age; in such a case, the levels of ligand-free receptor would be greater, leading to faster growth for an organism's age during the catch-up period [136]. Consistent with the *sizostat* hypothesis, Soliman and ElAwwa (2011) described that IGF-1R levels in the hypothalamus increase from neonatal to adult stages in mice [137]. The neuroendocrine hypothesis has been disputed by Baron and collaborators [138]. These researchers suggest that catch-up growth and recovery after a local perturbation is a cell-autonomous process and reflects an intrinsic property at the growth plate (the growth plate hypothesis), without any action of endocrine signals or cross-organ communication between the left and right side. In the growth plate hypothesis, chondrocytes are assumed to have a pre-determined proliferative potential and produce a fixed number of progenitors. The model postulates that after a slow growth condition, the affected limb would grow faster (‘catch-up' growth) because their chondrocytes would be developmentally younger [138]. Recent studies have supported the alternative view that undamaged chondrocytes indeed can sense the perturbation and counterbalance it for promoting bilateral limb symmetry [22]. These findings also support the Tanner's hypothesis, and the extended neuroendocrine hypothesis [9, 22, 137] and support the existence of inhibitor factor(s) acting as part of a feedback mechanisms that that stabilize organ size and body symmetry similar to those found in flies (see below).

In insects, the abnormal (e.g. a tumour) and defective growth of an imaginal disc influences the growth of other imaginal discs [139–140]. For instance, surgical elimination of a single or a pair of imaginal discs revealed that perturbed imaginal discs produce an inhibitory signal that slows down the growth of unperturbed imaginal discs [139, 140]. It was proposed that this inhibitory mechanism might act in the periphery and involve competition for a growth signal [140]. Early work also defined that tumour and injuries to the imaginal discs also influenced the neuroendocrine system, resulting in delayed maturation in insects e.g. [141, 142], similar to the observations in children and other animals [5, 9]. Recent studies also indicate that injured, and slow growing, imaginal discs inhibit the growth of the unaffected discs [143–145].

In mammals, the major hormones that regulate growth include growth hormone (GH), IGFs, glucocorticoids (GC), and thyroid hormones and, during the adolescent growth spur, the sex steroid hormones [9, 48, 136]. In insects, on the other hand, the juvenile hormone (JH), the insulin-like/IGF pathway and the steroid hormone ecdysone modulate juvenile development, growth rate, growth duration, and regenerative growth after tissue damage [45, 46, 146] (**Figure 2**). There is also evidence suggesting that these systemic controls not only act permissively but also provide instructive roles on how much or how little a structure or organ should grow. Hence, genomic or environmental variations that affect phenotypic outcomes such as bilateral asymmetry are expected to act in part, by affecting the production of such hormones or their responses in the periphery.

For example, numerous substances in the environment, food, and consumer products interfere with hormone biosynthesis and such endocrine disruptors can result in accelerated maturation, earlier growth cessation and ultimately shorter adult height [147]. Endocrine disruptors are also correlated with an increased risk of developing adult-onset diseases [148]. Studies in insects also support the notion that ultimately differences in size between the left and right sides or differences in body size induced by environmental factors (e.g. nutrition) involve extrinsic control of growth. For example, in insects, modulation of the JH has been associated with all known polyphenisms — alternative phenotypes (e.g., body size) that are induced by the environment (e.g., [139, 149]. The insulin/IGF and target of rapamycin (TOR) signalling provide a link between nutrition and growth control [45, 93]. Circulating IGFs (called Dilps in flies) are sensitive to numerous other environmental stresses, injury, and infection [150]. Moreover, variations in insulin/IGF signalling or response in growing tissues of an individual are both responsible for body size variation [151]. This is also evident in the exaggerated growth of weapons and ornaments of sexual selection [152] and the anomalous left-right overgrowth in asymmetric growth syndromes [33]. It has also been argued that slow growth compensation in response to growth perturbation is related to low levels of the steroid hormone ecdysone [17, 18, 128]. Others argue that dynamic stabilization of growth against perturbations requires multiple adjustments of growth hormones, maturation hormones and growth regulators [16, 153, 144, 153–155].

### Communication between the periphery and the brain: Dilp8-Lgr3 relaxin signalling

The *dilp8* gene was recently identified as an inhibitory feedback signal produced by growth perturbed imaginal discs in fruit flies [14, 15]. The Dilp8 signal, which is a new member of the insulin-like/IGF/relaxin family [14], acts on the neuroendocrine system in the brain [16–18]. During normal larva development and growth, *dilp8* is expressed in the growing imaginal discs at low levels. The *dilp8* expression levels decline even further as the larvae approaches its target size, but are then upregulated during metamorphosis [14, 15], and reviewed in [156]. If there is a growth perturbation (injury, slow growth, tumour) during larval growth, *dilp8* is acutely activated and secreted by the perturbed cells. This causes a delay in maturation that is proportional to the amount of growth to be recomposed and simultaneously downregulate the growth of the unperturbed parts to compensate for the extended growth period [14, 15]. In the absence of growth perturbations, expression of *dilp8* via a transgene is sufficient to induce the developmental arrest checkpoint and the slow down growth compensation, ultimately ensuring that correct target size and symmetry are attained despite extended growth period.

The *dilp8* gene is a strong candidate for the long-sought hormone that stabilizes growth across the body to ensure robust body size, symmetry and proportionality [14]. Flies deficient for *dilp8* have highly variable body sizes (interindividual variability) and disproportionate body parts, with some flies having larger than normal wings and other flies having smaller wings (**Figure 2A**). Furthermore, *dilp8* mutants have increased left-right bilateral asymmetry as measured by the fluctuating asymmetry index [14] and can show numerous morphological inaccuracies (**Figure 2A-B**: our unpublished observations). Variability in body size and bilateral asymmetry is increased even further by temperature and chemicals that induce DNA damage (**Figure 2C**).

Dilp8 binds to, and activates, a relaxin leucine-rich repeat-containing G protein–coupled receptor called Lgr3. Lgr3 activation mediates developmental homeostasis and size stabilization through a cyclic AMP-dependent pathway [16]. The Lgr3 receptor is required in two pairs of symmetric neurons within the central brain [16–18] (**Figure 2D**) and the prothoracic gland [154]. Larvae that lack *lgr3* in neurons do not respond to Dilp8 and importantly, exhibit high levels of fluctuating asymmetry [16–18].

The fact that neuronal Lgr3 is essential for keeping body and organ growth in check highlights that if the brain was unable to detect mismatches in growth, an organism would lose its ability to attain normal size and perfect bilateral symmetry. These discoveries that Dilp8 acts as a messenger of growth information (e.g., a growth deficiency) from the periphery to the brain in a defined set of neurons expressing Lgr3 (**Figure 2D**), not only represented a breakthrough in the field but also provides experimental supports for a neuroendocrine control of body size canalization [20]. The *dilp8* gene is activated in response to local disturbances and it then slows the growth of 'undisturbed' tissues. An analogous paracrine and systemic control also stabilizes the growth of the limbs of mice after injury [22], supporting that body symmetry and size may be controlled by universal mechanisms, although the specific factors may be distinct in different animals and/or peripheral tissues. Fifty years ago, it was postulated that individual tissues and organs could regulate their specific size by growth-inhibitory signals called *chalones*. These hypothetical tissue-specific mitotic inhibitors would be produced by each growing tissue in proportion to its mass (reviewed in [9]). The concept proposes that the regulation of growth is based on a negative feedback mechanism. Several molecules of the TGFβ family, including GDF8 (or Myostatin) and GDF11, have been identified as negative growth regulators with shark properties [9, 158]. Myostatin/GDF8, for example, keeps muscle size under control and its elimination produces animals with a dramatic increase in skeletal muscle mass. The loss of *dilp8* does not result in organs with excessive growth or larger animals. In contrast, Dilp8 acts to stabilize size in the face of growth perturbations and to ensure the target size is achieved. As such, loss of *dilp8* causes increased (size) variance not changes in mean size (scheme in **Figure 2A**).

How do Lgr3-responding neurons buffer variation? Recent studies have shown that Dilp8-mediated activation of Lgr3 signalling in the brain delays maturation and slows growth by co-regulating two neuronal populations, the PTTH-producing neurons [16, 17] (blue neurons in **Figure 2D**), and the insulin-producing cells (red neurons in **Figure 2D**) [16]. PTTH neurons project to prothoracic gland, a part of the endocrine ring gland, and regulate ecdysone production therein [159, 160]. However, this modulation alone is insufficient to adjust growth and stabilize body size [16, 128, 153]. Dilp8-activated Lgr3 also appears to balance growth by inhibiting the synthesis of JH and by lowering the amount of two IGFs, Dilp3 and Dilp5, produced by the insulin-producing cells (IPCs) of the brain [16]. Lgr3 signalling in the ring gland may also modulate the production of nitric oxide (NO) via NO synthase (Nos) [161], which could act as inhibitor signal for growth by among other functions suppressing DNA synthesis and reducing cell proliferation and by coordinating metabolism and maturating timing via the nuclear receptor E75 [161]. There are some complications for size control through NO/Nos. NO has a short half-life and thus is thought to act locally. Additional there are conflicting data on whether NO signalling promotes or inhibits growth and whether it affects or not developmental timing [126, 161, 162].

Although the proximate mechanisms that stabilize size in the imaginal discs are still unclear, one firm candidate is the transcription factor FOXO [163]. The transcription factor FOXO negatively modulates tissue growth and is a central hub of multiple signalling pathways that regulate cell growth, differentiation, and survival including the insulin/IGF signalling pathway, the JH and ecdysone signalling in response to many perturbations and in the protection against stress stimuli [35, 164, 165] and these characteristics make FOXO a candidate of size and symmetry regulation in the imaginal discs. Indeed, systemic activation of Dilp8 in the absence of perturbations triggers the developmental checkpoint and slows down the compensatory growth response associated with regulation of FOXO activity in imaginal discs [14, 16].

### Dilp8 as a hub of growth perturbation

The identification that *dilp8* is activated in response to an array of diverse growth perturbations (tumour, injury/regeneration, and slow growth etc.) that are induced by distinct mutations and oncogenes, environmental factors (e.g., radiation and DNA damaging agents), mechanical stress, and other stresses such as UPR stress [14, 15, 109, 166–171] suggests an universal mechanism for how the organism detects and manages local growth disturbances.

However, outstanding questions remain such as how the brain can compare the normal growth of the deviant one and adjust the levels and time of production of the hormones that regulate growth rate and maturation time, ensuring that each body part attains the correct size in relation to other parts and the whole body. Furthermore, *dilp8* activation does not appear to discriminate between over- and undergrowth. For example, *dilp8* is up-regulated in imaginal discs carrying ‘*Minute'* mutations [14, 15]. ‘*Minute'* mutations are caused by the haploinsufficiency of ribosomal genes and in flies and humans result in undergrowth and developmental defects [172]. *Minute* mutant cells activate the cell competition process in which fitter cells eliminate the slower growing, less-fit cells [112] and delay maturation [143]. These data may suggest that organ quality by cell competition may also involve mechanisms dependent on the Dilp8 signalling [14,15].

Numerous growth regulator and stress pathways, possibly in response to different growth perturbations, are known to activate expression of *dilp8* gene. These include the JNK [15, 109, 166], Notch [14], JAK/STAT [167], and Hippo/Yki pathways [19]. Various epigenetic factors may also play a role [14, 168, 169]. Furthermore, it is known that *dilp8* mRNA stability is negatively regulated by the XRN1 exonuclease [173].

Recently, an enhancer region of *dilp8* gene that directly responds to Yki/Scalloped has been characterized. This demonstrated the contribution of Hippo/Yki in developmental stability and control of left-right symmetry [19]. Deletion of this enhancer does not preclude the response of *Dilp8* to other perturbation stimuli, thereby suggesting that additional enhancer regions regulate Dilp8 expression in different contexts. Although Yki can induce transcription of *dilp8*, Dilp8 is not a classical Hippo/Yki target like *Diap1, ban* or *CycE* [19, 174]. In fact, it has been suggested that Yki may regulate expression of *dilp8* via interactions with the hormone regulator Taiman (Tai) [175]. Yki interacts with Tai to regulate programmes that are required for tissue overgrowth, programmes that might normally be suppressed by the Hippo/Warts pathway. In this way, Yki would only regulate Dilp8 during abnormal growth conditions and in a Tai/EcR-dependent manner [19, 175].

## BUFFERING FACTORS

Size robustness clearly entails mechanisms that ensure the correct expression and maintenance of key growth-related genes, even in fluctuating environments and despite unavoidable genomic variation. From a systems perspective, it can be argued that such robustness is a property of the entire network in which any disruption to key nodes or links can destabilize development by impairing network performance [24, 32]. Robust regulation of organ and body size requires mechanisms that mitigate (buffer) unavoidable variation in developmental pathways and factors regulating growth. Mutations can destabilize development, resulting in more variable than wild type phenotype [31, 176]. Here, we highlight the role of the main classes of factors known to contribute to developmental buffering during cell and animal development. For specific examples and further theoretical and experimental advances see [176–181] and further factors can be uncovered by genome-wide screen using chromosomal deficiencies and classical screens in multicellular organisms [182]. As some factors with a demonstrated role in filtering noise and environmental/mutational variation may also be important for protecting genome stability, our classification as ‘defences' and ‘buffers' is only for convenience.

### Asymmetric flies and cyclin G

The reproducibility of *Drosophila* wing size requires the *cyclin G* gene [41]. Cyclin G is a member of the family of atypical cyclins [183] encoded in mammals by the *CCNG1* gene and a conserved target of p53 [184]. Cyclin G cellular functions include growth regulation, cellular response to stress, DNA repair, genome stability, and the regulation of transcription, translational and epigenetic modulation (e.g., [177, 185–187]. Loss of *cyclin G* significantly enhances fluctuating asymmetry between the wings (reviewed in [177]). Curiously, asymmetric flies also arise from the overexpression of a short cyclin G isoform lacking the C-terminal PEST-rich domain (cycGΔP) [41]. Normally, organ size is robustly maintained by coordination and compensatory mechanisms between cell size and cell number [188]. Wings with overexpressed *cycG*Δ*P* have smaller cells and a reduced number of cells [189], whereas the inactivation of *cyclin G* by RNAi, on the other hand, results in the larger cells and increased cell number [187]. Thus, this mechanism is clearly impaired in asymmetric *cyclin G* mutant flies [41] and this explains much of the asymmetry in *cyclin G* flies [177].

### Cyclin G and genomic stability

Although numerous proteins are known to physically interact with cyclin G [187], the downstream effectors responsible for mediating developmental stability are only now beginning to be unveiled [189]. Cyclin G modulates cellular and organismal growth through insulin/IGF-like signalling due to its interaction with the B' regulatory subunits of PP2A, Widerborst and Well-rounded. These regulatory subunits negatively regulate Akt1 [190, 191], and important regulator of cell, organ, and organism growth [192]. Cyclin G also modulates Notch signalling, which is another important pathway in intrinsic growth control [45, 46, 193], through the recruitment of Hairless [194], a negative regulator of Notch [193].

Another interaction of interest is the conserved interaction between cyclin G and p53, which was first described in mammals [184, 195]. Ionizing radiation (IR)-induced DNA damage and other genotoxic stresses that cause DNA breaks are of particular threat to chromosome stability [196]. *Drosophila* cyclin G is a cofactor of p53 for the repair of double-stranded DNA breaks in somatic cells [197]. This finding raises the possibility that fluctuating asymmetry in *cyclin G* mutant flies might in part be due to unchecked p53-dependent DNA repair. Further studies are needed to determine the role of p53 and repair and surveillance mechanisms in the observed asymmetry of cyclin G and Dip8-Lgr3 deficient flies.

### Epigenetic regulators and buffering

Epigenetic control regulates cellular memory, such as stable maintenance of cell fate, and alterations in this control can cause non-genetic but heritable changes in gene expression. Interestingly, in *Saccharomyces cerevisiae*, an unbiased screen for genes conferring robustness to phenotypic variation found that deletion of H2A.Z and its chaperon SWR1 increases noise and phenotypic variation [54, 68]. H2A.Z is particularly interesting because it controls transcriptional efficiency and the transcriptional response to environmental factors [49, 58]. Yeast mutants lacking H2A.Z display high molecular and phenotypic noise (as reviewed in [187, 198]). Additionally, H2A.Z not only physically interacts with many proteins but also genetically interacts with many genes via epistasis. Proteins with these characteristics are generally predicted to confer buffering capacity [54, 68].

*Drosophila* studies have also linked the buffering of phenotypic variability with epigenetic control. For example, cyclin G physically interacts with Corto and Asx, two epigenetic regulators which act as enhancers of the Trithorax (Trx) and Polycomb (Pc) proteins [185, 186, 199] and imparts developmental stability, organ size precision, and reduces developmental noise via interactions with Polycomb Repressive Complexes [189]. Pc and Trx are involved in the maintenance of epigenetic gene activation and repression, respectively [200]. Furthermore, Trx is directly link to tissue damage-induced regenerative responses [168, 201]. It acts as a key regulator of *dilp8* expression during the developmental arrest checkpoint induced by regenerating tissues [166, 167] and thus potentially links cyclin G to Dilp8 [177]. Other buffering factors discussed in the following sections also linked developmental buffering to epigenetic regulation.

### The molecular chaperone Hsp90, buffering, DNA repair, and epigenetic reprogramming

The *Drosophila* molecular chaperone heat-shock protein 90 (Hsp90) was the first factor identified to participate in developmental buffering [105]. Since then, several groups have found that compromised activity of molecular chaperones and Hsp90 (encoded by *hsp83* in flies) — whether due to mutations, pharmacological inhibition, or temperature stress — unmask cryptic genetic variants that result in morphological variability in animals, plants and unicellular organisms (e.g., [105, 176, 202–204]). Pioneering studies in flies have shown that reduced levels of Hsp90 result in a high level of phenotypic variation among progeny. This variation is dependent on the genetic background and, when enriched by selection, causes ‘fixed' (i.e., independent of Hsp90 inactivation) phenotypic variants [176].

Hsp90 assists with the folding or unfolding of more than 200 “client” proteins that are involved in a wide range of processes, including cell-cell communication, organ-organ communication and organ construction [107], steroid and growth hormone receptors [205], and proteins associated with the DNA damage response, repair and chromatin remodelling [206]. The phenotypic diversity of impaired Hsp90 animals is likely a reflection of Hsp90 client diversity and the various developmental processes in which the client proteins are involved [176]. Hence, it is striking that bilateral symmetry in flies is not affected by Hsp90 [203, 207, 208] and see also considerations of the role of Hsp90 in developmental buffering [209]. Nonetheless, other researchers have found that reduction of Hsp90 function decreases developmental stability in other organisms [210, 211].

Takahashi *et al.* (2010) studied non-hsp90 heat-shock genes using transgenic RNA interference in *Drosophila* [212]. In this study, they uncovered a role for Hsp22, Hsp67Ba, Hsp67Bb, and Hsp67Bc in the developmental stability of bristle numbers. Furthermore, they found that the silencing of *hsp67Ba* increases both fluctuating asymmetry of bristle numbers and interindividual variation of wing shape, albeit only in males. However, as the RNAi line targeting Hsp67Ba has several off-targets, these results need to be taken with caution until further confirmation is obtained using independent RNAi lines or endogenous mutations [212]. These studies suggest that buffering stochastic noise and environmental and genetic variation requires the participation of different chaperones and heat-shock proteins. While the exact buffering mechanisms of these small heat-shock proteins is still unknown [212], they likely act via different, but partially overlapping pathways.

Recent studies also show that reduced activity of Hsp90 and other chaperons may not only expose pre-existing cryptic genetic variants of Hsp90 clients, but also generate phenotypic variability through impaired proper DNA repair and increased genomic instability [206], chromatin remodelling and epigenetic regulation of gene expression [213–216] and *de novo* induced mutations by transposon de-repression in the germline (see below).

### Transposon defence: the Hsp90-PIWI-piRNA pathway in the germline

Transposable elements make up approximately 5% of the euchromatic genome of *D. melanogaster* [217–219] and are responsible for 50-80% of spontaneous mutations in this organism [220]. Transposable elements are broadly classified as DNA transposons, which move to another location by a ‘cut-and-paste' mechanism, and retrotransposons, which are inserted into new locations by a “copy-and-paste” involving a reverse transcription of RNA intermediates and replication, hereafter both refers to as transposons. Although transposon insertions influence the evolution of the genomes, they are generally detrimental to the host organism and their accumulation decreases fitness [221, 222]. Transposon mobilization can generate deleterious mutations and gross chromosomal rearrangements through ectopic recombination [223] and so organisms have evolved conserved defence mechanisms that silence transposon activity.

*D. melanogaster* targets transposons in germline and somatic cells via two distinct RNA classes, the PIWI-interacting RNAs (piRNAs) [224, 225] and the endogenous siRNAs (esiRNAs or endo-siRNAs) [226–228]. The piRNAs are the largest class of small non-coding RNAs [229] central to the epigenetic and post-transcriptional silencing of retrotransposons in germline cells and also in some somatic cells [230–231]. PIWI-piRNAs were first discovered in flies but various piRNAs and PIWI-related pathways have now been involved in transposon silencing in various vertebrate species e.g. [232].

Recent studies have suggested that phenotypic variation in animals with reduced Hsp90 activity may originate from *de novo* mutations induced by transposons. Specchia *et al.* linked Hsp90 activity with the PIWI-piRNA pathway [233] and observed that individual flies with impaired Hsp90 activity have several transposons mobilized at new sites within their genome. They found a specific phenotypic variant to be linked with the *de novo* insertion of an I*-*element-like transposon sequence within a gene called *noc* [233]*.* They suggest that a reduction in Hsp90 activity may relax transposon silencing in germline cells and thus generate population diversity through *de novo* transposon-induced mutations. In this way, Hsp90 might act not only as a buffer but also as a defence of genome stability.

The PIWI protein forms a protein complex with Hsp90 and its co-chaperone Hop, the Hsp70-Hsp90 organizing protein [234], and prior work have shown that Hsp90 controls small non-coding RNAs. Consistently, not only PIWI itself, but also the Hsp90 or its co-chaperone Hop, are required for supressing phenotypic variation [234]. Moreover, Karam *et al.* (2017) also demonstrated that the co-chaperone Hop is required in the germline for silencing transposons [235]. Based on these data, the PIWI, Hsp90-HOP complex and small non-coding RNAs are critical for silencing transposon in the germline and regulation genome integrity.

Transposon silencing also involves the transcription factor p53 [238]. In human cancer cells, geldanamycin, which is a drug that inactivates Hsp90, causes the degradation of the p53 protein [107, 236, 237], thereby suggesting that p53 is a client of Hsp90. This potentially links p53 with Hsp90 and the PIWI-piRNA pathway in developmental buffering. Indeed, recent studies have shown that p53 restricts the mobilization of retrotransposons in germline cells via the PIWI-piRNA pathway [238].

piRNAs are transmitted maternally and, consistently with maternally derived mutational or epigenetic effects, only female (and not male) mutants of the PIWI-piRNA pathway show Hsp90-dependent increased phenotypic variation [213, 234]. Altogether, these studies uncover different mechanisms behind Hsp90-associated phenotypic variation and highlight a role for both transposons and epigenetic reprogramming.

### Transposon defence: endo-siRNAs

Endo-siRNAs are readily distinguishable from piRNAs [226, 239–241]. In *Drosophila*, they are produced by endogenous double-stranded RNA substrates and are almost always 21 nucleotides long [227]. Impairing endo-siRNAs in the germline or somatic cells does not impair piRNAs [228], indicating complementary roles in transposon silencing. Whereas PIWI-piRNA loss causes sterility owing to de-repression of transposons in the germline, factors involved in endo-siRNA-specific production suggest endo-siRNAs may play a role in transposon defence in somatic cells and the male germline ([228]: citation therein). As such, piRNAs and endo-siRNAs likely have distinct contributions to phenotypic variation/robustness. In addition, endo-siRNAs also defend genome integrity against exogenous nucleic acids such as viruses [242, 243].

The role of individual endo-siRNAs is still largely unknown. However, in *Drosophila*, mutations in the two *Dicer* genes uncouple the biogenesis of microRNAs from those of siRNAs [244]. The endo-siRNA silencing pathway requires Dicer-2 (Dcr-2) [241]. Unlike loss of *dcr-*1, which results in embryonic lethality, loss of *dcr-2* in flies as in *C. elegans* results in adults that are mostly normal and fertile [245]. This suggests that the endo-siRNAs are dispensable for major developmental processes. Curiously, however, in null *dcr-2* mutant flies, a fraction of endo-siRNAs are still generated by a yet unknown mechanism [227]. Since their recent discovery in *Drosophila*, a number of studies have provided examples suggesting that endo-siRNAs may be used for buffering variation in response to various stresses [178]. For example, the embryonic segmentation gene network is highly robust and compensates for different sources of noise such as temperature stress [246, 247]. While *dcr-2* mutant embryos are developmentally normally in unstressed conditions, *dcr-2* mutant embryos produce highly abnormal segmentation patterns during temperature stress [248]. Other studies have also shown that *dcr-2* mutants render animals more sensitive to other stresses [249].

Some endo-siRNAs may also directly or indirectly regulate specific mRNAs in a manner that is similar to the regulation of endogenous coding genes by miRNAs by pairing to their target mRNAs [250]. In this regard, the predicted mRNA targets of fly endo-siRNAs include stress response genes [240, 251]. The expression profile of RNA and protein in *dcr-2* mutants supports a role for endo-siRNA in the cellular response to stress [252]. Studies of the heat-stock response also indicate that endo-siRNAs are necessary for regulating expression of heat stress-related genes under unstressed conditions [253]. Thus, endo-siRNAs exert different roles within somatic cells, including both transposon silencing and genome protection against viruses. Nonetheless, future investigations of endo-siRNAs are needed to clarify their role in developmental stability.

### MicroRNAs and buffering of stochastic noise and environmental stress

miRNAs are a major class of non-coding molecules being investigated for their role in the buffering of gene expression and protein concentration levels that arise from stochastic noise and environmental perturbations [254–256]. miRNAs repress endogenous messenger RNAs (mRNAs) by pairing to seed sequences within the 3'-untranslated region (UTR) [257, 258]. This repression is generally classified as tuning or buffering e.g. [259–261]. Often, the knockouts of buffering miRNAs only produce a phenotype under certain genetic [262–264] or stress conditions [260, 265–267]. Several reviews have focused on miRNAs as buffers [178, 254].

Here, we briefly review how miRNAs buffer genetic noise. Many miRNAs act in feedback and incoherent feed-forward loops [256, 268] which are widespread strategies for reducing noise in developmental systems [24, 51, 58]. An incoherent feed-forward loop motif, for example, can involve a transcription factor regulating both the miRNA and its target gene [269]. This type of motif is attractive for noise attenuation because transient and random increases in transcription factor activity that would result in increases in target mRNA transcription are cancelled out by simultaneous increases in miRNA. This type of feed-forward loop enables protein output to be decoupled from fluctuations in transcriptional noise [59].

miRNAs can also mitigate the effects of environmental stresses and by helping restore homeostasis by modulating genes involved in the stress response [265, 271–274] and repair [275]*.* Furthermore, there are also well-characterized examples of miRNA reducing the effects of genetic mutations [262, 263]. Stress can also modulate the activity of miRNAs by reducing specific activities of the proteins involved in its biogenesis [267].

Although miRNAs and other systems strategies implicated in filtering noise have not yet been shown to affect body (a)symmetry, their role in body symmetry and developmental stability is anticipated since genes causing wing asymmetry, such as the proapoptotic gene *hid* [52] are target genes of robust-promoting miRNAs (e.g. miR-263a/b [264]). Additionally, miRNA might also contribute to systemic roles during developmental buffering by targeting genes in the biosynthesis of endocrine and systemic growth factors [276, 277–282]. Moreover, as in mammals, certain miRNAs in *Drosophila* are released by tissues and circulate in the haemolymph [282] and such secretory miRNAs may act not only within the cell and organ where they are expressed but also at remote sites [283]. Nonetheless, empirical validation of secretory miRNAs in flies is still lacking and one study to date has demonstrated strict cell-autonomous activity for specific target mRNAs [284]. Future studies on specific miRNAs under stress conditions are needed to elucidate their function in developmental stability and intraindividual precision and bilateral symmetry.

## CONCLUDING REMARKS AND OPEN QUESTIONS

Symmetric growth is a fascinating but often overlooked phenomenon of biology. The processes during development which help to ensure two sides of a body are perfectly matched are still poorly understood. Such precision needs to overcome not only significant variability in individual cell proliferation, growth rate, number and death [3, 5, 50], but also both genomic and environmental variation. For this purpose, cells within growing organs use a variety of redundant strategies and quality controls to select the fittest cells and attain the correct target size in the face of perturbations. However, as low but continual accumulation of DNA damage and mutations can overwhelm editing systems, buffering mechanisms are essential for mitigating the effects of such unavoidable variation.

Our understanding of the complex relationships between biological noise, environmental and genomic variations and phenotypic invariance (or plasticity) is still incomplete. From a systems biology view, robustness reflects the fidelity and efficiency of developmental regulatory networks [32] and here, we have highlighted studies of specific factors that may impart robustness to these networks and autonomously act at the cell and organ level. Genetic screens for factors involved in fluctuating asymmetry have been performed using chromosomal deficiencies e.g. [182] and although well-known buffers such as Hsp90 was not identified, we had noted that the screen identified deficiencies uncovering factors in the Dilp8-Lgr3 signalling. Some of the characterized ‘buffers' might also play important roles in defending genome stability against external and internal damaging factors and thus compromising the activity of these factors (e.g. cyclin G, hsp83/Hsp90, PIWI, etc.) may increase mutational load by reducing the fidelity of DNA repair mechanisms and by relaxing transposon silencing. While the role of *de novo* somatic mutations in cancer is well characterized, their contribution to non-cancerous disease and phenotypic variability remains less explored. Future genomic studies of body asymmetry and genetic screens may help to isolate genes important to resist to somatic mutations.

Recent studies also point to genes that may promote developmental precision and body symmetry acting systemically. Here, we have discussed the importance of the relaxin hormone Dilp8 and its receptor Lgr3 in the stabilization of body size, proportion and symmetry both in response to environmental and genetically-induced growth perturbations and under physiological, and apparently absence of external perturbations. Thus, *dilp8* and *lgr3* mutants can capture the impact of environmental or genetic variation and the effect of stochastic cellular noise in body size and symmetry. Hence, through systems, genetic and molecular approaches, these mutant flies offer an entry point to identify further mechanisms and factors underlying somatic cell resilience and sensitivity to stochastic noise, and/or genomic and environmental variation.

In the early 1960s, Tanner postulated the neuroendocrine or *sizostat* hypothesis to account for how children catch up and regain their normal growth after a variety of growth conditions and illnesses [20]. An extended Tanner's hypothesis also account for how children and other animals may recover from local injuries. A neuroendocrine control of body size and symmetry suggests that a central mechanism continuously monitors body size (and mismatches) and accordingly makes corrective adjustments to ensure correct target size is achieved. Recent studies support the systemic control and feedback inhibitory mechanism that may stabilize growth in the face of perturbation in insects and vertebrates [14, 16–19, 22]. Importantly, a recent work of catch up growth in fetal mice using a novel mosaic approach to manipulate unilaterally gene expression unveils that stabilization of bone growth requires compensatory growth and coordination of growth involving IGF signaling within and between organs like in flies [22]. These findings can serve as an entry point to further understand how size and symmetry remain undeterred in noisy environments, after injury or illnesses, and in the presence accumulated somatic mutations. In the future, this information may have broad implications for fitness and health.

## Abbreviations:

ER: endoplasmic reticulum
IGF: insulin-like growth factor
JH: juvenile hormone
JNK: Jun-N-terminal kinase
NO: nitric oxide
Trx: Trithorax
UPR: unfolded protein response
